# Biomedical Holistic Ontology for People with Rare Diseases

**DOI:** 10.3390/ijerph17176038

**Published:** 2020-08-19

**Authors:** Laia Subirats, Jordi Conesa, Manuel Armayones

**Affiliations:** 1 Eurecat, Centre Tecnològic de Catalunya, C/Bilbao, 72, 08005 Barcelona, Spain; 2 eHealth Center, Universitat Oberta de Catalunya, Rambla del Poblenou, 156, 08018 Barcelona, Spain; jconesac@uoc.edu (J.C.); marmayones@uoc.edu (M.A.)

**Keywords:** biomedical ontologies, medical health records, interoperability, sentiment analysis

## Abstract

This research provides a biomedical ontology to adequately represent the information necessary to manage a person with a disease in the context of a specific patient. A bottom-up approach was used to build the ontology, best ontology practices described in the literature were followed and the minimum information to reference an external ontology term (MIREOT) methodology was used to add external terms of other ontologies when possible. Public data of rare diseases from rare associations were used to build the ontology. In addition, sentiment analysis was performed in the standardized data using the Python library Textblob. A new holistic ontology was built, which models 25 real scenarios of people with rare diseases. We conclude that a comprehensive profile of patients is needed in biomedical ontologies. The generated code is openly available, so this research is partially reproducible. Depending on the knowledge needed, several views of the ontology should be generated. Links to other ontologies should be used more often to model the knowledge more precisely and improve flexibility. The proposed holistic ontology has many benefits, such as a more standardized computation of sentiment analysis between attributes.

## 1. Introduction

In Europe, a disease is called rare when it has an incidence of fewer than five cases out of every 10,000 inhabitants [1]. There are some 7000 known rare diseases, which, according to the World Health Organization (WHO) estimates, affect 7% of the world’s population. The magnitude of the problem is huge, given that these pathologies are characterized by early onset (two out of three of the pathologies appear before the age of two). In addition, one in five patients suffers from chronic pain, and the development of motor, sensory or intellectual abilities has deficits in half of the cases. Finally, in almost half of the cases, a vital prognosis is at stake, since rare diseases have a 35% deaths rate before one year, 10% between one and five years and 12% between five and fifteen years.

The conditions involved in rare diseases are huge, complex and heterogeneous. There is also little knowledge about them due to the few cases; the complexity and the number of conditions involved; and the lack of shared natural histories. Therefore, some mechanisms to facilitate the integration of medical data would be beneficial. In this sense, interoperability is a huge problem in rare diseases, as it is in medicine, which requires solutions on at least at three different levels: terminologies, ontologies and archetypes (examples of the latter are HL7 or CEN/ISO EN13606). In this paper, we focus on the first two. Since terminologies may be seen as lightweight ontologies [2], from now on, we use the term ontologies for referring to both ontologies and terminologies.

There are several biomedical ontologies that can be used to model patient histories; some of the most popular (according to their number of visits in Bioportal) are: Systematized Nomenclature of Medicine—Clinical Terms (SNOMED CT) [3], Medical Subject Headings (MESH) [4], Orphanet Rare Disease Ontology (ORDO) [5], International Classification of Diseases version 10 (ICD) [6], the International Classification of Functioning, Disability and Health (ICF) [7], Diabetes Mellitus treatment (DMTO) [8,9,10], Alzheimer’s disease (ADO) [11], Parkinson’s disease (PDON) [12], Multiple sclerosis Ontology (MSO) [13] and Physical Medicine and Rehabilitation ontology (PMR) [14].

From a theoretical point of view, the existent biomedical ontologies can be seen as domain ontologies [15], since they describe the vocabulary related to general domains or tasks. The combination, and potential extension, of different biomedical ontologies to represent the information necessary to deal with a given problem (the rare disease *Amyopathic dermatomyositis* in European countries, for example) may be seen as an application ontology [15] (since it represents the concepts related to *Amyopathic dermatomyositis* in a given context and cannot be reused as is in other contexts). Another common characteristic of application ontologies is that they usually contain instances that represent the data related to the context of interest. Different needs may require different application ontologies, and consequently, different combinations and refinements of the current biomedical ontologies. Therefore, agile methodologies to evaluate the quality of application ontologies in the biomedical context during its creation (or when they are created) are necessary. These methodologies allow guaranteeing that the created ontologies are complete, sound and interoperable.

Besides, there are other more recent taxonomies promoted by the World Health Organization (WHO) that could be useful, such as the International Classification of Health Interventions (ICHI). Furthermore, there are also impact assessment techniques [16] promoted by the WHO such as quality-adjusted life years (QALYs) and disability-adjusted life years (DALYs). Additionally, in rare diseases, several research initiatives map genetics with therapeutic target validation, such as [17], or put together both common and rare diseases, such as [18].

It should be highlighted that there are some initiatives sponsored by the World Health Organization (WHO) which promote a more holistic treatment of people or the study the bio-psycho-social states of the people by using, for example, the International Classification of Functioning, Disability and Health (ICF).

Social networks may also been considered, since they may have positive (or negative) effects in healthcare, as explained in [19]. Some benefits are that both patients and stakeholders have more information with social networks but at the cost of some risk, such as confidentiality loss or inaccurate information. There are several initiatives for personalization of ehealth in social media. One of those is in the smoking cessation area [17], showing that social media can help people to quit smoking. Some initiatives study how social media are aligned with the priorities of rare diseases associations [20]. Additionally, other initiatives focused more on Youtube videos, such as [21], which found that 29.3% of anorexia-related videos showed pro-anorexia behaviors. Other research initiatives use Twitter to predict the population health index, also using spatial big data [22]. Another initiatives study the impact of crowd-sourcing and friend-sourcing in Alzheimer disease [23].

For our use case, and to add information about the interactions of patients with virtual and physical environments, we propose to consider other non-medical ontologies, such as Semantically-Interlinked Online Communities (SIOC) [24] and LinkedGeoData (these ontologies are found via *Linked Open Vocabularies* [25]).

As aforesaid, there are several potentially useful ontologies to be considered. They may be useful when used alone, but it is only when they are integrated with other ontologies and data that they achieve their full potential [26]. This integration process and the validation of its result is a great challenge in the biomedical context, as [27] states. Apart from the elements commented on by [27], we believe that the integration should also consider non-biomedical information, comprising, at least, social network, geographical, geopolitical, temporal and demographic information.

The research question of the paper is: How can one use an application ontology that provides a holistic view of the patients and their diseases with a combination of current domain ontologies (biomedical and not) and data extracted from other sources? The proposal also helps in the creation of the application ontology since it determines what kind of information should be included and how it should be specified.

To do so, we analyzed what information is required to provide a holistic view of patients and their diseases, how to integrate it with existing biomedical (and generic) ontologies and how it covers the necessary information. Then, an application ontology that covers the information of the analyzed information was created and evaluated by analyzing its usefulness.

## 2. Materials and Methods

This section is divided into two subsections: The first section (rare disease scenarios) describes the analysis of over 50 rare diseases scenarios. The last section describes the methodology used to built the application ontology and the sentiment analysis library used to analyze its data. It should be highlighted that this study has been approved by the Ethics Committee of the Universitat Oberta de Catalunya on 15 January 2020 under the project identification code “Biomedical Holistic Ontology for People with Rare Diseases”.

### 2.1. Rare Diseases Scenarios

Regarding the description of scenarios related to people with rare diseases, we have analyzed 53 different scenarios extracted from different sources: Eurordis, The voice of Rare Disease Patients in Iran and the Spanish Federation of Rare Diseases (FEDER). Details from these scenarios have not been presented to preserve their anonymity. From these scenarios, some belong to top-ranked countries in overall health system performance according to the ranking of the health systems of the 191 member states of the World Health Organization (WHO) [28]; and some belong to the lowest-ranked countries according to this ranking. In addition, people interested in rare diseases of social networks have also been considered. Therefore, from the selected Tweets, the following data shown in Table 1 have been considered.

As previously mentioned, 53 scenarios were analyzed; however, not all of them contained information of interest. Therefore, from them only 25 testimonials have been modeled: seven testimonials from Eurordis, three testimonials from The voice of Rare Disease Patients in Iran and 15 testimonials from the Spanish Federation of Rare Diseases (FEDER). The main attributes collected are summarized in Table 2.

From the previous scenarios, we can see that information of the disease is necessary, but so is non-medical information such as environmental factors, activities, and interests. The non-medical data provides context to patients, but also to symptoms and conditions, and may facilitate a better comprehension of each patient, potentially allowing one to find out new information about his interaction with the disease. Examples of this are the inference of new symptoms of a given disease, the psychological impact of sharing and commenting in social networks, the evaluation of the differences of the emotional impact of the disease in different countries (with a better/worse health service or having more/fewer daylight hours per day) and the analysis of sentiment associated with the posts of patients together with their locations.

### 2.2. Methodology to Build the Ontology and to Perform the Sentiment Analysis

The methodology to build the ontology was bottom-up [29], starting with more specific classes obtained from the dataset, and afterwards, they were grouped in more general concepts. This methodology was chosen among top-down and mixed top-down and bottom-up methodologies because we wanted to represent with a minimum overhead the concepts of the dataset. In addition, Minimum Information to Reference an External Ontology Term (MIREOT) [30] plugin has been used with the ICF to import external terms to an ontology. Best practices of ontologies have been followed, and the ontology without imports of other ontologies have been validated with the software tool Oops! (OntOlogy Pitfall Scanner!) (http://oops.linkeddata.es) [31].

Finally, usefulness ontology has been evaluated by applying sentiment analysis over its data. The goal of this analysis is to identify the feeling of the patient when describing the different aspects of his experiences. Regarding content analysis, the sentiment analysis was performed using the Python library Textblob. In sentiment analysis both the polarity and the subjectivity were analyzed, and more attention was paid to the correlations of numeric variables. TextBlob Python library was backed up on Pattern with the Natural Language Toolkit (NLTK). This toolkit uses statistical approaches and regular expressions to determine the polarity and subjectivity of a given text. The values are based on the adjectives of the text and they are optimized based on the frequency of the adjectives and successive words. The sentiment analysis library returns two attributes when analyzing a text: polarity and subjectivity. The polarity score is a float within the range [−1.0, 1.0], where −1.0 is a negative text, 0 is a neutral text and 1.0 is a positive text. On the other hand, subjectivity is a float within the range [0.0, 1.0], where 0.0 is very objective and 1.0 is very subjective. Google Translate was used to translate the data from Spanish to English.

## 3. Results

We have created an ontology, named Holistic Ontology of people with Rare Diseases (HORD), which is available under the license http://purl.org/NET/rdflicense/cc-by4.0 without instances of people with rare diseases information in Bioportal. (The locations of the linked ontologies (ICD and SIOC) had to be changed in the created ontology and uploaded to https://github.com/laiasubirats/rarediseasesontology since these ontologies were not accessible at their specified locations). The ontology uses links to other ontologies using plugin MIREOT when possible (MIREOT was used in the ICF ontology).

**Figure 1:**
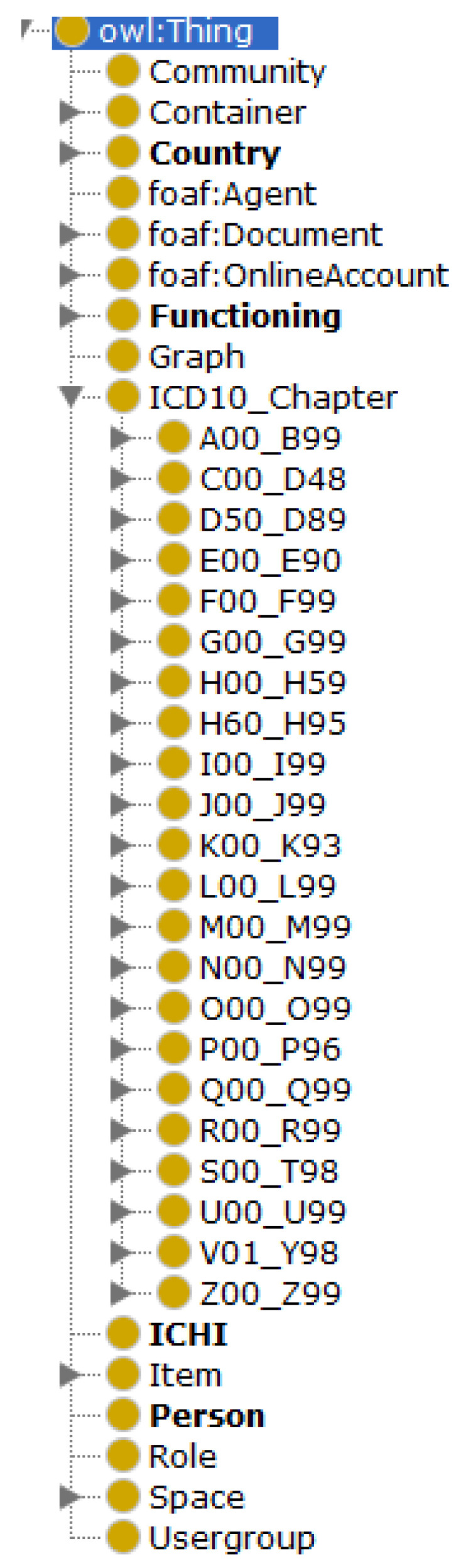
Class diagram of the proposed ontology.

**Figure 2:**
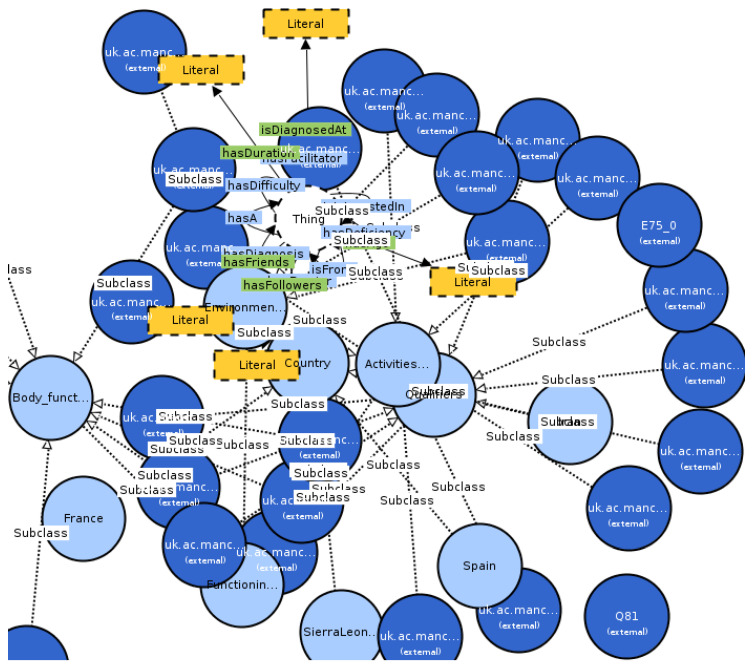
Partial Visual Notation for OWL Ontologies (VOWL) representation of the proposed ontology.

The resultant ontology imports the external ontologies ICD (medical information) and SIOC (contextual information) and has more than 14,000 concepts and 80 individuals that represent the analyzed scenarios. The result of the Oops! validation has been that there are not important nor critical pitfalls. Figure 1 shows the main classes of the ontology and Figure 2 shows some of its main classes and relationships graphically.

Regarding Figure 2, we can see in different colors a fragment of the ontology HORD. Each circle represents a class of the ontology. The color of the circle depends on the type of class. Lines are used to represent property relations, and arrowheads indicate the directions of the property relations by pointing to the objects of the properties. Arrowheads representing range axioms are filled with the foreground color; arrowheads representing subclass relations are filled with the neutral color. Property labels and data types are shown in rectangles. If representing data types, the rectangles have the data type color and a border. If representing property labels, they are without a border and colored according to the property type. The lines and borders of some types of classes and properties are dashed or dotted. A dashed line indicates set operators and class disjointness (if visualized). A dashed border indicates literals and special types of classes. A dotted line is reserved for subclass relations.

The metrics of the ontology are the following:

- Number of classes: 14,558;
- Number of individuals: 85;
- Number of object properties: 69;
- Number of data properties: 32;
- Total classes MIEROTed: 27;
- Maximum depth: 4;
- Maximum number of children: 35;
- Average number of children: 7;
- Classes with a single child: 11;
- Classes with more than 25 children: 6.

In addition, it should be considered that the description logic expressivity of the HORD ontology is SHI(D) which is the symbol key of:

- S: An abbreviation for ALC with transitive roles.
- AL: Attribute language. This is the base language which allows: atomic negation (negation of concepts that do not appear on the left-hand side of axioms), concept intersection, universal restrictions and limited existential quantification (restrictions that only have fillers of things).
- C: Complex concept negation.
- H: Role hierarchy (subproperties: rdfs:subPropertyOf).
- I: Inverse properties.
- (D): Use of datatype properties, data values or datatypes.

**Figure 3:**
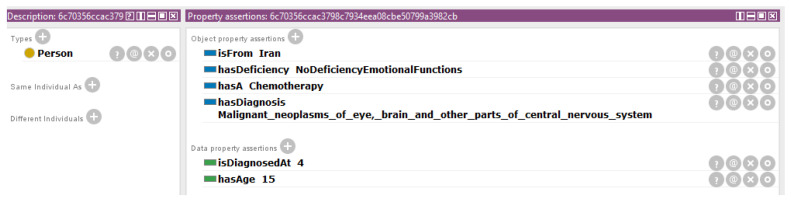
Anonymous representation of a patient in the proposed ontology. A SHA1 operation has been performed on the names of the people in order to preserve their anonymity.

**Figure 4:**
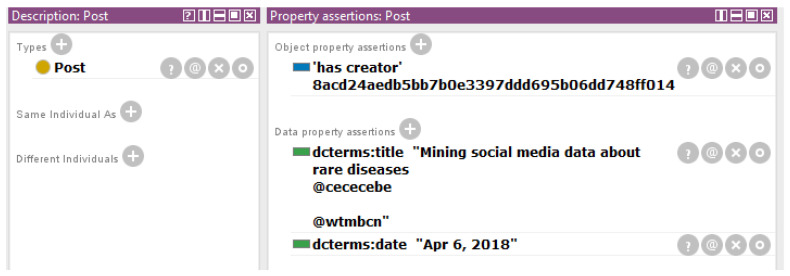
Anonymous representation of a post of a person interested on rare diseases in the proposed ontology. A SHA1 operation has been performed on the names of the people in order to preserve their anonymity.

To model the scenarios, the recommendations of ICF standard have been followed. In addition, to model diseases with ICD-10 the webpage Orphanet has been used because some of the rare diseases are not available yet at ICD-10 (however, more of them will be available at 2018 at ICD-11 [32]). For example, the Wolfram Syndrome is modeled with Orphanet as “E13.8 Other specified diabetes mellitus with unspecified complications”. The modeling of an anonymized scenario is shown in Figure 3 and the modeling of a Twitter scenario is shown in Figure 4. It should be considered that a SHA1 algorithm (http://www.sha1-online.com) has been applied to the names of the people in order to preserve their anonymity.

Regarding sentiment analysis, both the polarity and subjectivity of the code have been made available at the GitHub repository. In Table 3, the main correlations between polarity and subjectivity and numerical variables were computed from data extracted from the scenarios. In bold are highlighted the absolute correlations equal to or above 0.3. In Table 3, we can see that the higher absolute correlations are for age of diagnosis-polarity, age of diagnosis-subjectivity, emotional functions-polarity and remunerative functions-polarity. It makes sense that emotional functions and remunerative employment have negative correlations, as the ICF standard establishes complete deficiency/difficulty (4), severe deficiency/difficulty (3), moderate deficiency/difficulty (2), mild deficiency/difficulty (1) and no deficiency/difficulty (0). Since few scenarios are represented in the example, no further analyses are meaningful. However, in a real world environment, it would be interesting, for example, to take into account the diseases to find out the more negative aspects of each diseases or to cluster diseases according to the aspects that generated discomfort to their patients. These kind of analyses cannot be done when contextual and medical data are not integrated.

## 4. Discussion

The implications of the findings of this research study could lead to other research studies including a more holistic view of validating ontologies and including more relationships between different areas, boosting interdisciplinary studies. On the other hand, this research has several socioeconomic impacts: research, society, quality of life, economic and generation of code impact.

Regarding the impact on research, the new ontology can be used in other domains and can enhance and promote other domains to be more holistic. In addition, gathering all the medical and contextual data together may help to understand and facilitate patients’ diagnoses and develop new therapies for rare diseases.

As for the impact on society, the generated data and the correlations between attributes can help people to understand their behavior and have a better knowledge of the situations of people with rare diseases. In addition, the system is open and uses well-established standards, providing a natural platform for knowledge sharing related to rare diseases.

Quality of life is not only how a person feels physically. It is also essential how the person feels psychologically and socially. This study helps people with rare diseases and their stakeholders to use social networks to improve their quality of life.

With reference to economic impact on rare diseases, rare diseases are usually forgotten by health systems and pharmaceuticals. This study helps to give visibility to rare diseases and the reality they face and the differences between countries. This study helps both administrations and hospitals to care more about rare diseases and to help them with important aids, such as the law of dependency.

Finally, the code is openly available in a GitHub repository.

However, this study has also some limitations. When making the application of the ontology broader, scalability can become a problem. Ontologies may become less manageable, meaning that scalability problems appear when using tools such as Oops!, as processes are slower. Furthermore, evolution of machine-translation systems is moving fast and providing higher quality year after year, as can be seen in as [33], which analyzes the use of automatic translation systems and its impact in sentiment analysis, and [34], which studies the usefulness of comparative research of automatic translation systems regarding different languages. However, we are aware of the validity issues that may arise from the use of automatic translation of scenarios from Spanish to English in our case. Finally, the authors performed a manual validation over the data to test the efficiency of the Textblob library, obtaining a correct polarity of 20 out of 25 texts. Manual validation involved three people: two of them evaluating each history by hand and the third one resolving the discrepancies. However, it must be highlighted that texts were rather long (some of them over 900 words) and they had mixed feelings, which made it difficult for the Textblob library to extract polarity.

## 5. Conclusions and Future Work

This research has fulfilled the following objectives:

- A new holistic ontology about rare diseases has been built and shared. This ontology was composed by the integration of existing ontologies (medical and contextual) and includes information about 25 scenarios of people with rare diseases. The ontology has been validated and usefulness assessed. Depending on the user (a patient, a health professional, a policy maker, etc.) some parts of the ontology may be more interesting than others; thus, several views of the ontology should be generated.
- Code is shared openly to the community so that this research is partially reproducible.
- People are informed about the importance of supporting rare diseases and the problems of this collective. It is an objective to disseminate this study in Biomedical repositories such as Bioportal in order to inform the general public about problems involved in rare diseases. Therefore, these efforts aim to engage other people to work in this domain, helping the collective and providing it with more information.

As future work, a testimonials dataset could be openly shared with the explicit consent of all users. In addition, the inclusion of these validation rules could be more integrated with reviewing tools such as the one provided by Bioportal. Another possible line of research is finding out the best way to treat missing reviews, and how to aggregate those reviews. Another possibility is some study about how the reputations of users could affect the validation of the ontology. On the other hand, we will address the effectiveness of Google Translate, which is the system we used to reduce the cost of translation, in further work. Finally, in further work, we propose to use aspect-based sentiment analysis to identify the different topics dealt with in each history (cost, diagnosis, pain, disability, public health, etc.) and the polarity for each topic.

## Figures and Tables

**Table 1:** Data considered in the Tweets.

Attribute	Description
Time	UTC time when a Tweet was created
Geo_coordinates	Represents the geographic location of a Tweet as reported by the user or client application. The inner coordinates array is formatted as geoJSON (longitude first, then latitude)
User_lang	Language used in the Tweet
SHA1 (Secure Hash Algorithm 1) of In_reply_to_user_id_str	If the represented Tweet is a reply, this field contains the string representation of the original Tweet’s author ID. This will not necessarily always be the user directly mentioned in the Tweet.
SHA1 of In_reply_to_screen_name	If the represented Tweet is a reply, this field contains the screen name of the original Tweet’s author.
In_reply_to_status_id_str	If the represented Tweet is a reply, this field contains the string representation of the original Tweet’s ID.
SHA1 of From_user_id_str	Identifier of the user who authored the Tweet
User_followers_count	Count of the followers of the user
User_friends_count	Count of the friends of the user
User_location	Location of the user
Entities_str	Hashtags, indices and other information of the user

**Table 2:** Attributes collected in the scenarios.

Category of Attribute	Attributes
Demographic and clinical information	Name, age, country, disease, age of diagnosis and treatment.
Body functions	Emotional functions, consciousness, vomiting, respiratory functions, skin functions, hearing and vestibular functions, cognitive functions, and pain in head and neck.
Activities and participation	Interests, remunerative employment, non-remunerative employment, higher education, sports, arts and culture, and walking.
Environmental factors (facilitators and barriers)	Technological facilitators for communication, barrier regarding health professionals, barrier in financial assets, and barrier in health systems.

**Table 3:** Correlations between some numerical attributes and polarity and subjectivity of the testimonial.

Attribute [min, max] Mean (std)	Polarity	Subjectivity
Age [1, 45] 23 (11.2)	−0.15	−0.02
Spain [0, 1] 0.7 (0.5)	0.13	−0.01
Iran [0, 1] 0.1 (0.3)	−0.12	−0.23
Age of Diagnosis [0, 31] 9.3 (8.2)	**−0.31**	**0.40**
Emotional Functions [0, 4] 0.7 (1.0)	**−0.47**	−0.07
Remunerative employment [0, 4] 0.7 (0.7)	**−0.30**	0.01

## Abbreviations

The following abbreviations are used in this manuscript:

ADO	Alzheimer’s disease
DALY	Disability-adjusted life year
DMTO	Diabetes Mellitus treatment ontology
FEDER	Spanish Federation of Rare Diseases
HL7	Health Level Seven International
HORD	Holistic Ontology of people with Rare Diseases
ICD	International Classification of Diseases
ICF	International Classification of Functioning, Disability and Health
ICHI	International Classification of Health Interventions
MESH	Medical Subject Headings
MIREOT	Minimum Information to Reference an External Ontology Term
MSO	Multiple Sclerosis Ontology
NLTK	Natural Language Toolkit
OOPS	OntOlogy Pitfall Scanner
ORDO	Orphanet Rare Disease Ontology
PDON	Parkinson’s disease
PMR	Physical Medicine and Rehabilitation Ontology
QALY	Quality-adjusted life year
RD	Rare disease
SHA	Secure hash algorithm
SIOC	Semantically-Interlinked Online Communities
SNOMED CT	Systematized Nomenclature of Medicine—Clinical Terms
WHO	World Health Organization
